# Snapchat filters changing young women's attitudes

**DOI:** 10.1016/j.amsu.2022.104668

**Published:** 2022-09-17

**Authors:** Ashna Habib, Tooba Ali, Zainab Nazir, Arisha Mahfooz

**Affiliations:** Dow University of Health Sciences, Mission Road, Karachi, Pakistan

Social media is being used by 4.48 billion people worldwide. Snapchat is one of these social media platforms that provides a wide range of filters and editing tools that let people change their photos to look like any version of themselves they can imagine. Over 6 Billion snaps are created every day on average as of the primary quarter of 2022, Snapchat has 332 million daily dynamic clients worldwide. The App Positions as the 12th Most Prevalent Social Media Stage Worldwide.Females are overwhelming the Snapchat stage, making up 54.4% of its clients.

Particularly women have been found to publish photos to social media more frequently than men do, as well as to update, manage, and maintain their personal profiles more frequently [1]yet, limited work has delved into how these particular images impact female viewers. Since these representations are less prevalent than those seen in mainstream media, it is crucial to comprehend how regular exposure to these expertly manipulated and heavily filtered photographs by this app causes ordinary women to become detached from reality. Additionally, people who are familiar with each other offline are possibly more inclined to notice differences between their peers' online and offline personas and criticize them for particular online activities.Research is required to better understand how women see other women who use filters and post on social media platforms, as well as if understanding of filter use helps mitigate the unpleasant views that exposure to these filters can cause in women.

Larger eyes, bigger lips, more angular jawlines, whiter teeth, and slimmer faces may all be achieved with the filters, as well as smooth and even skin tones. Additionally, users can crop, shade, and colorize. The way that young women see beauty has been greatly altered by these filters and editing tools.Several signs point to the alarming use of Snapchat filters and photo-editing practices among young women that the Young women alter their appearance in photographs to the point where their true selves no longer pass muster. They begin to see problems in their appearance that no one else even notices, such as the shape of their face or a broad forehead. They spend a lot of time taking and retaking selfies, then editing them to get the ideal shot. In order to cover up defects or imperfections, young ladies frequently glance through earlier photos. They do this, which causes them to feel unhappy, has a negative impact on their self-esteem, and is a source of aggravation. They ultimately desire to change their appearance because they believe that they should look the way they appear in the filtered version of themselves. This indicates that they are no longer able to analyze how they appear in reality. Young women frequently react by feeling self-conscious when they see a photo of themselves without any filters.

Self-presentation includes “changing and modifying the self throughout social encounters to generate a desired impression on the audience,” [2].Therefore, in order to provide a good first impression, social media users feel pressured to exhibit the most appealing versions of themselves to others [2].However, these photographs frequently don't represent a person's genuine physical appearance [2].Over the past few years, the occurrence of patients asking for surgeries to resemble their selfie or filtered picture has been referred to as “Snapchat dysmorphia” [3] In the near future, “Snapchat dysmorphia” might continue to be a developing trend and it will cause an increase in plastic surgery trends. Filters frequently cause despair, anxiety, unhappiness, and other mental health issues. Despite their best efforts, young women are unable to keep up with the realities of everyday life. They constantly strive to meet the high standards for beauty set by this world.The adoption of conventional beauty standards as portrayed in the media is the primary contributor to self-objectification in young women that leads to appearance anxiety.

Filters are distorting one's perception of oneself and are creating unreasonable and unnatural expectations of one's appearance. When these expectations are not met, this comparison leads to stress, appearance dissatisfaction, sensitivity to feedback and judgments about appearance, negative self-esteem, and low mood. They will need to be discussed more frequently, especially among Millennials. Millennials are predicted to take 25,000 selfies on average over their lifetimes [4]Repeated interactions with filtered images and associated beliefs and worries are increasing the risk of mental health issues such as depression or social anxiety, and issues such as appearance anxiety including thoughts like excessive worrying and behaviors like appearance checking and cloaking.There hasn't been a lot of quantitative study connecting the use of photo-editing apps and Snapchat filters with young women's attitudes.

For its young users, Snapchat's filters are setting off an endless cycle of highs and lows. Socially minded patient education should be considerate of those who are troubled by comparative social media's negative impacts, such as the usage of edited and filtered images and the importance of continual feedback. Such a study is required because appearance anxiety leads to behaviors depicting appearance-related worries rather than just how filters affect people's perception of their bodies, which can limit daily activities and obstruct constructive social connections. In order to close the research gap, further studies should be conducted to determine how the use of photo-editing applications like Snapchat filters is connected to young women's changing habits.

Young women report higher feelings of appearance anxiety than males do, and their anxiety levels tend to rise more over adolescence [5]. Snapchat filters and depression are connected but further studies are required to pinpoint any potential causes as women are more likely than men to experience depression. This is especially true given the likelihood that visual interactions and comparisons of appearance between young people increase as new such applications and platforms are introduced to the market. Future studies should examine the efficacy of limiting daily filter use and raising literacy levels in lessening the burden of mental health issues among young women. Finally, it's important to comprehend both the immediate and long-term impacts of using filters.

## Ethical approval

No ethical approval required for this paper.

## Funding

This article did not receive any specific grant from funding agencies in the public, commercial, or not-for-profit sectors.

## Author contributions

**Ashna Habib:** conception of study, major drafting of work, literature search, final approval and agreeing to the accuracy of work.

**Tooba Ali:**conception of study, major drafting of work, literature search, final approval and agreeing to the accuracy of work.

**Zainab Nazir:**conception of study, major drafting of work, literature search, final approval and agreeing to the accuracy of work.

**Arisha Mahfooz:** conception of study, major drafting of work, literature search, final approval and agreeing to the accuracy of work.

## Registration of research studies

No patients were included.

Name of the registry:

Unique Identifying number or registration ID:

Hyperlink to your specific registration (must be publicly accessible and will be checked):

## Guarantor

Ashna Habib, Tooba Ali, Zainab Nazir, Arisha Mahfooz.

## Consent

This study was not done on patients therefore no written consent was required.

## Declaration of competing interest

Authors state no conflict of interest.
